# Evaluation of the UP4FUN Intervention: A Cluster Randomized Trial to Reduce and Break Up Sitting Time in European 10-12-Year-Old Children

**DOI:** 10.1371/journal.pone.0122612

**Published:** 2015-03-31

**Authors:** Frøydis N. Vik, Nanna Lien, Sveinung Berntsen, Ilse De Bourdeaudhuij, Monika Grillenberger, Yannis Manios, Eva Kovacs, Mai J. M. Chinapaw, Johannes Brug, Elling Bere

**Affiliations:** 1 Department of Public Health, Sport and Nutrition, University of Agder, Kristiansand, Norway; 2 Department of Nutrition, University of Oslo, Oslo, Norway; 3 Department of Movement and Sport Sciences, Ghent University, Ghent, Belgium; 4 Department of Nutritional Behaviour, Max Rubner-Institute, Federal Research Institute of Nutrition and Food, Karlsruhe, Germany; 5 Department of Nutrition and Dietetics, Harokopio University, Athens, Greece; 6 Department of Pediatrics, Pecs University, Pecs, Hungary; 7 Department of Public and Occupational Health and the EMGO Institute for Health and Care Research, VU University Medical Center, Amsterdam, the Netherlands; 8 Department of Epidemiology and Biostatistics and the EMGO Institute for Health and Care Research, VU University Medical Center, Amsterdam, the Netherlands; University of St Andrews, UNITED KINGDOM

## Abstract

**Background:**

The UP4FUN intervention is a family-involved school-based intervention aiming at reducing and breaking up sitting time at home (with special emphasis on screen time), and breaking up sitting time in school among 10–12 year olds in Europe. The purpose of the present paper was to evaluate its short term effects.

**Methodology/Principal Findings:**

A total of 3147 pupils from Belgium, Germany, Greece, Hungary and Norway participated in a school-randomized controlled trial. The intervention included 1–2 school lessons per week for a period of six weeks, along with assignments for the children and their parents. Screen time and breaking up sitting time were registered by self-report and total sedentary time and breaking up sitting time by accelerometry. The effect of the intervention on these behaviors was evaluated by multilevel regression analyses. All analyses were adjusted for baseline values and gender. Significance level was p≤0.01. No significant intervention effects were observed, neither for self-reported TV/DVD or computer/game console time, nor for accelerometer-assessed total sedentary time and number of breaks in sitting time. The intervention group, however, reported more positive attitudes towards (β = 0.25 (95% CI 0.11, 0.38)) and preferences/liking for (β = 0.20 (95% CI 0.08, 0.32)) breaking up sitting time than the control group.

**Conclusions/Significance:**

No significant intervention effect on self-reported screen time or accelerometer-assessed sedentary time or breaks in sitting time was observed, but positive effects on beliefs regarding breaking up sitting time were found in favor of the intervention group. Overall, these results do not warrant wider dissemination of the present UP4FUN intervention.

**Trial Registration:**

International Standard Randomized Controlled Trial Number Registry ISRCTN34562078

## Introduction

It has been suggested that decreasing any type of sedentary time is associated with lower health risk in youth, independent from physical activity levels [1]. Sedentary behaviors include sitting and lying down [2,3], but not sleep. Because of the increased availability of electronic forms of entertainment, such as television (TV), DVD, computer activities and electronic gaming, more sedentary time has been observed among children [4]. Such sedentary activities have been associated with overweight, obesity and metabolic health [5,6]. However, to date there is insufficient evidence for a prospective association between sitting time and biomedical health [7]. Also, just a small number of studies have examined associations between children’s objectively assessed overall sedentary time and health outcomes [7,8].

A recent meta-analysis [1] and a systematic review [7] revealed that sedentary time among children is most often operationalized as TV time. TV watching in excess of 2 hours daily is associated with reduced physical and psychosocial health, and reducing sedentary time may lead to reductions in body mass index (BMI) [1]. TV time is also associated with more exposure to marketing of unhealthy foods, and possibly to unhealthier snacking [9–11]. In recent years computer time contributes more to total screen time; a recent study showed that computer time constituted more than one third of the total screen time among children across Europe [12]. The ENERGY study across 7 European countries reported that boys spend about 2½ hours/day and girls somewhat less than 2 hours on screen-viewing activities (TV and computer-time combined), and that mean total screen time, as well as TV and computer time, was higher for boys than girls [12].

Apart from lowering total screen- or sedentary time, breaking up prolonged sitting time may be especially beneficial [13]. Most studies on breaks in sitting time and associations with health outcomes are to our knowledge performed among adults. Healy et al. [14] found that independent of total sedentary time and moderate-to-vigorous intensity physical activity, increased breaks in sedentary time were positively associated with lower waist circumference and BMI. However, recently, a study among children was published by Saunders et al. reporting that breaks in sedentary time was associated with reduced cardio metabolic risk and lower BMI Z-score in both boys and girls with a family history of obesity [15]. A study by Kwon et al. [16], showed that breaks in sedentary time have notably decreased during childhood and adolescence (from 5 to 15 years of age), and that both boys and girls had fewer breaks in sedentary time during school hours than during any other period of the week or weekend.

Two meta-analyses of interventions among children found small, but significant effects on screen time reduction [17] and sedentary time reduction [18]. However, Salmon et al. pointed out that only few of the recent studies aiming to reduce TV viewing or sedentary time among young people have been very successful [8]. Reasons for this may possibly be that most studies have been conducted in only one setting (i.e. home based, school based or community based) and that intervening in multiple settings may be more effective [8].

In order to address screen time in interventions, insights in the potential determinants of these behaviors are necessary [4]. Quite strong and consistent evidence has been found for an inverse association between parental rules/restrictions regarding screen-based behaviors and sedentary behavior [19]. Further, a positive association between availability of screens (TVs and computers) and parental screen time has been reported [19].

The UP4FUN intervention aimed to reduce total screen time and increase breaking up sitting time at home (i.e. during screen time) and breaking up sitting time at school (i.e. in classroom sessions) among schoolchildren in different countries across Europe [20]. UP4FUN is part of the ENERGY project (EuropeaN Energy balance Research to prevent excessive weight Gain among Youth) [21], a European Commission funded project to study overweight and obesity and possibilities for prevention in 10–12 year old schoolchildren across Europe [21].

The ENERGY project aimed to extend, update and learn from earlier evidence on obesity prevention and apply this knowledge to contribute to obesity prevention across Europe, and consisted of two major parts [21]. The first part consisted of reviews, secondary data analyses, focus groups and a cross European school-based survey in seven countries conducted to compile and enrich the existing evidence regarding obesity and potential determinants of obesity in 10–12 year olds. The second part of the ENERGY project consisted of the systematic development, implementation and evaluation of an intervention (i.e. the present UP4FUN intervention) in order to further develop the evidence base for prevention of childhood overweight and obesity [21].

The present study evaluates whether the UP4FUN intervention had an effect on reducing screen time and increasing breaking up sitting time (primary outcome), and also in changing specific personal determinants for these behaviors (e.g. awareness, attitude, self-efficacy regarding sedentary activities) and family environment determinants (e.g. parental practices, social environment, physical environment) (secondary outcome) among children and their parents in five countries across Europe.

## Materials and Methods

### Development and content of the UP4FUN intervention

The UP4FUN intervention was systematically developed, based on the five steps of the Model of Planned Promotion for Population Health [22] and guided by the Intervention Mapping Protocol [23]. The protocol for this trial and supporting CONSORT checklist are available as supporting information; see S1 CONSORT Checklist and S1 Protocol. The intervention was developed in a socio-ecological framework [24] due to the influence of the home physical and social environment, with strategies targeting the child, family and school [21,25]. Changing personal determinants of sedentary time (e.g. awareness, attitude, and self-efficacy regarding sedentary activities) was considered important to promote self-regulation, because children in this age group are likely to spend some amount of non-supervised time at home. The taxonomy of behavior change techniques by Abraham and Michie [26] was applied to characterize the link between the determinants and intervention components.

The name UP4FUN, referred to standing up and finding fun alternatives to sedentary activities. UP4FUN included a school component, a child component and a family component with all pupils within an intervention school receiving the UP4FUN intervention. The school component of the intervention included one or two lessons (45 minutes each) per week and lasted for six weeks. Project members trained the teachers prior to the intervention. Teachers were also given a teacher manual including the outline of each lesson and were provided with material to be handed out to the children. Each week focused on particular topics, week 1: introduction to UP4FUN, week 2: increasing awareness about sedentary behaviors, week 3: goal setting related to sedentary behavior, week 4: influence of the home environment on sitting time, week 5: breaking up prolonged sitting time and practicing active transportation to school and week 6: summary of the UP4FUN intervention. The child component included assignments during school classes or at home. Examples of intervention activities for the children during the six weeks were: registering sitting time, registering steps with a pedometer, making a list of fun non-sedentary activities, writing personal goals to reduce sitting time, evaluating personal goals, writing down difficulties regarding achieving their goal and proposing solutions, writing down the number of children in class with rules about screen time and some examples of the rules, discussing family screen time rules, brainstorming ideas for non-sedentary recess activities and making a poster, two minutes activity breaks per sitting lesson, motivation to try the activity breaks at home and encouragement to practice active transportation to school. The family component included six newsletters, one per week/theme in colorful paper-designs, which were handed out by the teacher to the children to bring home to their parents. The newsletters contained personalized messages from the children and homework tasks to be completed at home, sometimes by the children and the parents together. The intention of the newsletters was to involve the parents in the intervention. Incentives were used to support motivation and enhance the fun part of the intervention (pedometers and stickers), as well as the social commitment to the project (bracelets with UP4FUN-logo). Further details of the development of the UP4FUN intervention have been described elsewhere [20].

### Study design

In order to evaluate the intervention, it was implemented in autumn 2011 in a school-randomized trial with a pre- and post-test design. Randomization by school, instead of pupils, was the only feasible method of conducting UP4FUN and reporting therefore complies with the Consort 2010 statement: extension to cluster randomized trials [27]. “Clusters” are in this study schools, and “participants” are children/pupils. Pupils in their final years of primary education (first years of secondary education in Germany) (aged 10–12 years) from Belgium, Germany, Greece, Hungary and Norway, i.e. in different regions of Europe participated, as well as one of every child’s parents. The control schools were asked to continue with their usual school curriculum. A convenience sample (i.e. in the proximity of the participating institutions) of schools was chosen. For practical reasons a convenience sample was chosen in order to make the evaluation of the intervention feasible within a limited time frame. Clusters eligible for inclusion were schools with at least two classes with children in the required age group to ensure a large enough sample size. A sample size of 2500 children (i.e. 500 in each country) has been indicated by previous studies of school-based interventions to be sufficient to detect effects of such an intervention [21]. This was confirmed by new power calculations based on screen time data (self-reported) from the ENERGY cross sectional survey indicating that this sample size would be sufficient to detect a 20% decrease in total screen time. Schools were invited by a letter followed by a phone call, and sometimes a parent meeting to explain the nature and purpose of the intervention. The schools were recruited before the summer holiday (May-July), and randomized upon final recruitment. Parental consent was asked after randomization; i.e. at the beginning of the school year (August/September) in both intervention and control schools. The schools were then paired according to size in order to get similar numbers of children in intervention and control groups. Then one school in each pair was randomly drawn to the intervention condition by the ENERGY-project coordinator from a country not involved in this evaluation study, while the remaining school was allocated to the control condition and was offered the intervention package after the post-test. All pupils age 10–12 years (and parents/caretakers) attending the recruited schools were invited to participate through an invitation letter. A pre-test pen and paper questionnaire survey was conducted in September/October 2011 among pupils and parents, and a similar post-test survey which included the same child and parent questionnaire as at the pre-test (with some extra process evaluation questions for the intervention condition) in November/December 2011. The questionnaires were completed during one school-lesson (45 minutes) by the pupils in the presence of a trained researcher. The survey was administered Tuesdays to Fridays in order to cover week-days only with 24-h recall questionnaire items. Finally, a subsample of 20% of the children in each country was asked to wear an accelerometer for one week before the intervention to obtain objective baseline data on sedentary time and breaks. After the post-test, accelerometer-data were collected for the second time among the same children. The accelerometer data from one of the intervention regions, Belgian Flanders, have already been published elsewhere [28].

### Study sample

**Fig 1:**
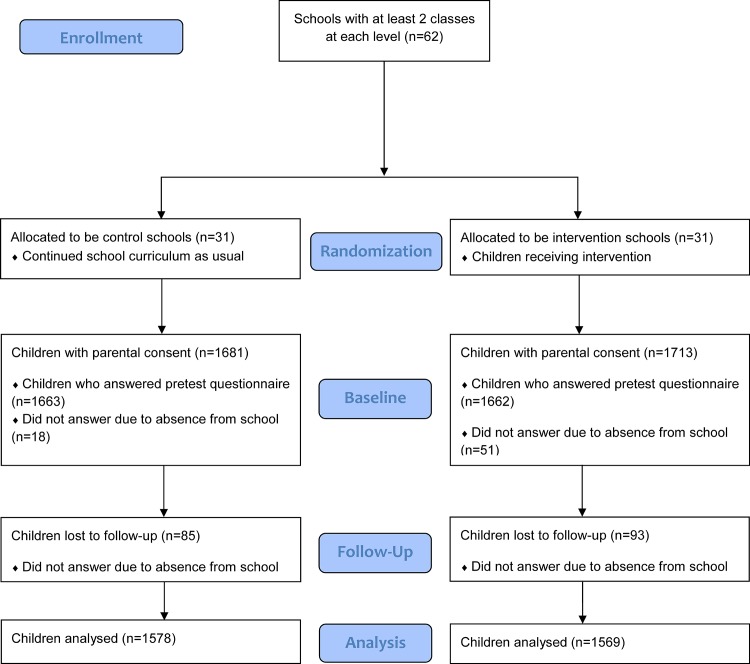
Flow Diagram of UP4FUN schools with participating children. Enrollment and randomization of schools, number of children at baseline and follow-up and number of children analyzed (intervention and control).

In total, 62 schools participated (participation rate 41%); 31 intervention schools (including 105 classes implementing the intervention) and 31 control schools (including 114 classes). In these schools there were 5117 eligible pupils, of which 3394 (66%) were given parental consent. Of these pupils, a total of 3325 pupils completed the pre-test survey, 3232 completed the post-survey and 3147 pupils completed both surveys (Fig 1). These 3147 pupils constitute the study sample for the present outcome evaluation; 1569 intervention pupils and 1578 control pupils. Out of the 3147 pupils, 584 wore accelerometers at pre-test or post-test due to limited availability of accelerometers. Of these, 150 wore accelerometers at both time points and had valid data, and thereby constitute the study sample for the accelerometer analyses.

### Instruments/Measures

All variables used to evaluate this study are presented in S1 Appendix. A questionnaire was used to assess screen time, breaking up sitting time and determinants of these behaviors. Primary outcomes: Screen time was assessed as hours per day of TV/DVD watching and computer/games console use reported by the children, and was based on measures of both (1) frequency (FQ), and (2) what they did “yesterday” (i.e. the day before the survey day, 24h-recall). The number of breaks from sitting time by the children during one hour of TV/DVD watching, one hour of computer/games console usage and one school hour, were reported in breaks/hour sitting and breaks/school hour, respectively. Secondary outcomes: Several potential determinants for TV/DVD watching, computer/games console usage and breaking up sitting time were also assessed in the child questionnaire. Most determinants were operationalized as statements, with five response alternatives ranging from I fully agree to I fully disagree. The response alternatives were coded from 2 (most positive regarding health) to -2 (most negative regarding health). A few other determinants were operationalized differently (e.g. as the number of TV’s at home).

The questionnaire was developed in English, translated into the relevant five languages, translated back into English and checked for misinterpretation. The screen time items (both on behaviors and determinants) and the items on socio demographic variables used in the UP4FUN study, were derived from the child questionnaire used in the cross sectional study of the ENERGY project [29]. The test-retest reliability and construct validity of the screen time measures and their determinants have previously been published [30], and these results indicate good test-retest reliability and moderate to good construct validity for the relevant items. The items on breaking up sitting time and the potential determinants of breaking up sitting time were developed by the research team for the present study, because such items did not exist since these issues were not researched before. The questionnaire, including these new items, was therefore pre-tested among 27 children across the five participating countries before the project started, in order to assess if they were feasible in our target group, and a separate test-retest reliability study was conducted to test these psychometrics for the newly developed items (S1 Appendix).

### Test-retest reliability study

A convenience sample of six schools (not participating in the intervention) was selected in autumn 2011; one school in Belgium, four schools in Hungary and one school in Norway. A total of 143 pupils (57% girls; participation rate 64%) from Belgium, Hungary and Norway completed the questionnaires at both time points (seven days apart). The test-retest reliability test showed moderate to good values for most items (ICC 0.51–0.96), except for the items on determinants of breaking up sitting time that showed poor to moderate test-retest reliability values (ICC 0.24–0.56) based on criteria from Nunnally and Bernstein [31]. The test-retest reliability data are presented in S1 Appendix.

### Accelerometer measurements

The short period to measure a large sample of children necessitated the use of all available accelerometer models by ActiGraph (7164, LLC, Fort Walton Beach, Florida, USA) in the research groups; (either GT1M, GT3X or ActiTrainer). All accelerometers were worn on the right hip, secured by an elastic waist belt. Accelerometers were initialized using ActiLife software [32], selecting a 15-s epoch measurement interval. Children were instructed to wear the accelerometer for seven consecutive days during all waking hours, but to remove it during bathing and other water activities. Minimal wear time was set at eight h/day (i.e. indicating a valid day), and the minimum number of valid days was four [33]. Non-wear time was defined as 60 minutes or more of consecutive zero counts/min. Total sedentary time was accumulated time below 100 counts/min across all valid days. Sedentary bouts were defined as a period of at least ten consecutive minutes in which accelerometer output was below 100 counts/min. Within these sedentary bouts, zero counts above 100 counts/min were tolerated. Breaks in sitting time were defined as transition in accelerometer count from 100 counts/min to >100 counts/min in between two sedentary bouts [34].

### Statistics

Test-retest reliability for the UP4FUN child questionnaire was assessed with agreement at the individual item level (S1 Appendix). Previously, the child questionnaire used in the cross-sectional survey within the ENERGY project, had been assessed in the same way [30]. The agreement of categorical items (mostly Likert-type scales), continuous, and dichotomous items was analyzed with a two-way random effects single measure intraclass correlation coefficient (ICC 2.1). Because the calculation of the ICC depends on the existence of the variability in answering categories, we also calculated percentage agreement.

Descriptive analyses of baseline values on differences in behaviors and determinants between intervention and control group were conducted using independent sample t-test (Tables 1–4). To assess the effects of the intervention, each behavior or determinant was analyzed in separate multilevel (i.e. adjusted for school as a random effect) regression models with post-test values of each behavior and each behavioral determinant as dependent variable, and condition as independent variable, adjusted for gender and baseline values, as recommended by Twisk and Proper [35]. Accelerometer data was also adjusted for total accelerometer wear time. To test a potential moderating effect of gender, an interaction term between condition (intervention vs. control) and gender was added to the model, but since this was not significant for any of the analyses, it was removed from all analyses. All different behaviors and determinants were also analyzed separately by country to investigate if there were any country-specific differences among the children regarding the items studied. With two behaviors (total screen time and breaking up sitting time) and 44 potential determinants of total screen time and 4 of breaking up sitting time, a total of 50 dependent variables were tested in 57 regression models. Because of multiple testing, the significance level was set at p≤0.01. All analyses were conducted using SPSS 19 (SPSS Inc. Chicago, IL).

**Table 1:** Baseline and follow-up values of self-reported determinants of TV/DVD.

**Determinants TV/DVD**	Baseline TV/DVD			Follow-up TV/DVD		Intervention effect Ω	
	Control	Intervention	p-value	Control	Intervention	β (95% CI)	p-value
**Personal determinants**							
Awareness	0.08 (0.02,0.15)	0.07 (0.01,1.13)	0.77	0.31 (0.22,0.40)	0.39 (0.30,0.48)	0.08 (-0.04,0.20)	0.21
Knowledge	1.83 (1.78,1.88)	1.79 (1.73,1.84)	0.23	1.81 (1.75,1.88)	1.84 (1.78,1,91)	0.03 (-0.06,0.12)	0.51*
Attitude	0.46 (0.40,0.52)	0.43 (0.37,0.50)	0.51	0.50 (0.40,0.61)	0.56 (0.46,0.66)	0.05 (-0.09,0.20)	0.45**
Preference/liking	-1.11 (-1.15,-1.06)	-1.11 (-1.16,-1.06)	0.94	-1.16(-1.23,-1.09)	-1.11(-1.18,-1.03)	0.06 (-0.05,0.16)	0.28
Self-efficacy	0.27 (0.20,0.34)	0.25 (0.18,0.32)	0.74	0.53 (0.44,0.61)	0.38 (0.29,0.46)	-0.16(-0.27,-0.03)	0.01
Automaticy	0.03 (-0.04,0.10)	0.04 (-0.03,0.11)	0.92	0.20 (0.10,0.30)	0.15 (0.06,0.25)	-0.05 (-0.18,0.09)	0.49
**Family environment**							
Parental practices§							
Parents let child watch	0.20 (0.13,0.27)	0.34 (0.26,0.41)	0.01	0.22 (0.13,0.31)	0.33 (0.24,0.42)	0.11 (-0.02,0.24)	0.10
Ask parents to watch, child allowed	-0.69 (-0.73,-0.64)	-0.62 (-0.66,-0.57)	0.03	-0.73(-0.78,-0.67)	-0.72(-0.78,-0.67)	0.00 (-0.08,0.08)	0.94
Parent rules on watching	0.37 (0.30,0.45)	0.39 (0.32,0.47)	0.73	0.24 (0.12,0.36)	0.36 (0.24,0.47)	0.17 (-0.05,0.28)	0.17
OK for child that parents have rules	0.96 (0.89,1.02)	0.97 (0.91,1.04)	0.73	0.89 (0.80,0.97)	0.87 (0.79,0.95)	-0.02 (-0.13,0.10)	0.74
Child take part in setting rules	0.60 (0.57,0.63)	0.57 (0.54,0.60)	0.09	0.64 (0.60,0.69)	0.68 (0.64,0.73)	0.04 (-0.02,0.10)	0.20
Parents remind about rules	0.62 (0.59,0.65)	0.57 (0.54,0.60)	0.01	0.65 (0.59,0.70)	0.64 (0.58,0.69)	-0.01 (-0.09,0.07)	0.85
Parents reaction—angry	0.10 (0.03,0.17)	0.05 (-0.02,0.12)	0.30	-0.20(-0.28,-0.11)	-0.18(-0.27,-0.10)	0.01 (-0.11,0.13)	0.85
Parents reaction—less friendly	-0.23 (-0.31,-0.16)	-0.30 (-0.38,-0.23)	0.20	-0.46(-0.55,-0.37)	-0.46(-0.55,-0.37)	0.00 (-0.12,0.13)	0.95
Parents reaction—explanation	0.94 (0.88,1.01)	0.95 (0.89,1.02)	0.82	0.89 (0.79,0.99)	0.85 (0.75,0.95	-0.05 (-0.19,0.10)	0.52
Social environment							
Parental modeling	-0.28 (-0.32,-0.24)	-0.28 (-0.32,-0.24)	0.93	-0.26(-0.31,-0.21)	-0.31(-0.36,-0.27)	-0.05 (-0.16,0.01)	0.12
Watching together with parents	3.54 (3.39,3.69)	3.39 (3.25,3.53)	0.17	3.52 (3.36,3.68)	3.52 (3.36,3,67)	0.00 (-0.23,0.22)	0.99
Parental subjective norm	0.85 (0.79,0.90)	0.92 (0.87,0.98)	0.07	0.86 (0.78,0.94)	0.92 (0.85,1.00)	0.06 (-0.05,0.17)	0.26
Physical environment							
Home availability—TV's	2.43 (2.38,2.49)	2.42 (2.37,2.48)	0.80	2.50 (2.47,2.54)	2.52 (2.48,2.56)	0.02 (-0.04,0.07)	0.51
Home availability TV bedroom	0.43 (0.41,0.46)	0.42 (0.39,0.44)	0.53	0.44 (0.42,0.46)	0.43 (0.41,0.44)	-0.01 (-0.04,0.01)	0.22

Unadjusted means and 95% confidence intervals for the baseline and follow-up values of self-reported determinants of TV/DVD, the results of independent t-tests comparing intervention and control groups (at baseline), and regression coefficients (beta’s) with 95% confidence intervals as results from multilevel multiple regression analyses (independent models for each behavior) testing the effects of the UP4FUN intervention.

Ω: adjusted for school (as a random effect), gender and baseline level.

§: see S1 Appendix for complete items in the child questionnaire.

*Significant for Hungary β = 0.22 (95% CI 0.08, 0.36), p = 0.003.

**Significant for Norway β = 0.26 (95% CI 0.10, 0.42), p = 0.001.

**Table 2:** Baseline and follow-up values of self-reported determinants of computer/games consoles.

**Determinants computer/ games consoles**	Baseline computer/games console			Follow-up computer/games console		Intervention effect Ω	
	Control	Intervention	p-value	Control	Intervention	β (95% CI)	p-value
**Personal determinants**							
Awareness	0.46 (0.39,0.53)	0.49 (0.43,0.56)	0.53	0.53 (0.45,0.62)	0.56 (0.48,0.64)	0.03 (-0.09,0.14)	0.65
Knowledge	1.52 (1.47,1.53)	1.52 (1.47,1.57)	0.86	1.62 (1.55,1.69)	1.59 (1.52,1.66)	-0.03 (-0.12,0.07)	0.59
Attitude	0.57 (0.51,0.64)	0.56 (0.50,0.63)	0.86	0.55 (0.45,0.65)	0.62 (0.52,0.72)	0.07 (-0.07,0.21)	0.32*
Preference/liking	-0.76 (-0.82,-0.69)	-0.72(-0.78,-0.65)	0.39	-0.83 (-0.92,-0.74)	-0.84 (-0.92,-0.75)	-0.01 (-0.13,0.12)	0.94
Self-efficacy	0.49 (0.41,0.56)	0.44 (0.36,0.51)	0.34	0.53 (0.45,0.61)	0.51 (0.43,0.60)	-0.02 (-0.14,0.10)	0.77
Automaticy	0.38 (0.31,0.45)	0.37 (0.30,0.44)	0.89	0.47 (0.38,0.54)	0.45 (0.36,0.54)	-0.03 (-0.15,0.10)	0.69
**Family environment**							
Parental practices§							
Parents let child watch	0.26 (0.19,0.33)	0.37 (0.30,0.44)	0.04	0.36 (0.27,0.44)	0.39 (0.30,0.47)	0.03 (-0.09,0.15)	0.60
Ask parents to watch, child allowed	-0.60 (-0.64,-0.55)	-0.51(-0.56,-0.46)	0.01	-0.57 (-0.63,0.50)	-0.57(-0.63,-0.50)	0.00 (-0.10,0.09)	0.94
Parent rules on watching	0.43 (0.35,0.51)	0.44 (0.36,0.51)	0.92	0.30 (0.18,0.41)	0.38 (0.26,0.50)	0.09 (-0.08,0.25)	0.31
OK for child that parents have rules	0.89 (0.83,0.96)	0.88 (0.81,0.95)	0.77	0.77 (0.66,0.88)	0.75 (0.65,0.86)	-0.02 (-0.17,0.13)	0.82
Child take part in setting rules	0.59 (0.56,0.62)	0.58 (0.55,0.61)	0.87	0.64 (0.61,0.68)	0.64 (0.61,0.68)	0.00 (-0.05,0.05)	0.94
Parents remind about rules	0.65 (0.63,0.68)	0.59 (0.56,0.62)	0.003	0.65 (0.59,0.71)	0.66 (0.61,0.74)	0.01 (-0.07,0.10)	0.75
Parents reaction—angry	0.01 (-0.07, 0.08)	-0.08(-0.16,-0.01)	0.08	-0.27 (-0.34,-0.20)	-0.28(-0.35,-0.21)	-0.01 (-0.11,0.09)	0.86
Parents reaction—less friendly	-0.25 (-0.32,-0.17)	-0.39(-0.46,-0.32)	0.01	-0.55 (-0.64,-0.46)	-0.46(-0.55,-0.36)	0.09 (-0.04,0.23)	0.16
Parents reaction—explanation	0.93 (0.87,1.00)	0.89 (0.82,0.95)	0.33	0.83 (0.73,0.93)	0.76 (0.66,0.86)	-0.07 (-0.21,0.07)	0.32
Social environment							
Parental modeling	0.26 (0.21,0.32)	0.26 (0.20,0.32)	0.95	0.25 (0.17,0.33)	0.28 (0.20,0.36)	0.03 (-0.08,0.15)	0.57
Watching together with parents	1.18 (1.07,1.29)	0.98 (0.88,1.08)	0.01	1.23 (1.08,1.38)	1.03 (0.88,1.17)	-0.20 (-0.41,0.01)	0.06
Parental subjective norm	0.91 (0.85,0.97)	0.98 (0.92,1.04)	0.08	0.89 (0.81,0.97)	0.97 (0.89,1,04)	0.08 (-0.03,0.19)	0.16
Physical environment							
Home availability number PC	2.41 (2.34,2.47)	2.44 (2.38,2.51)	0.44	2.53 (2.43,2.62)	2.53 (2.44,2.63)	0.01 (-0.12,0.14)	0.90
Home availability own PC	0.48 (0.45,0.50)	0.46 (0.44,0.49)	0.37	0.50 (0.48,0.52)	0.49 (0.47,0.51)	-0.01 (-0.04,0.02)	0.52
Home availability internet	0.95 (0.94,0.96)	0.94 (0.93,0.96)	0.38	0.94 (0.93,0.96)	0.95 (0.94,0.96)	0.01 (-0.01,0.02)	0.53
Home availability games consoles	2.15 (2.07,2.23)	2.15 (2.08,2.23)	0.90	2.29 (2.15,2.42)	2.37 (2.24,2.50)	0.08 (-0.10,0.27)	0.38
Home availability own games console	0.66 (0.64,0.68)	0.67 (0.65,0.69)	0.66	0.67 (0.64,0.70)	0.68 (0.65,0.71)	0.01 (-0.03,0.05)	0.66
Home availability cell phone	0.72 (0.70,0.75)	0.74 (0.72,0.76)	0.27	0.73 (0.71,0.75)	0.75 (0.73,0.77)	0.02 (-0.01,0.05)	0.17

Unadjusted means and 95% confidence intervals for the baseline and follow-up values of self-reported determinants of computer/games consoles, the results of independent t-tests comparing intervention and control groups (at baseline), and regression coefficients (beta’s) with 95% confidence intervals as results from multilevel multiple regression analyses (independent models for each behavior) testing the effects of the UP4FUN intervention.

Ω: adjusted for school (as a random effect), gender and baseline level.

§: see S1 Appendix for complete items in the child questionnaire.

* Significant for Hungary β = -0.39 (95% CI -0.57, -0.21), p<0.001.

**Table 3:** Baseline and follow-up values of self-reported and objectively measured behaviors of breaking up sitting time and self-reported determinants of breaking up sitting time.

**Behaviors and determinants breaking up sitting time**	Baseline breaking up sitting time			Follow-up breaking up sitting time		Intervention effect Ω	
	Control	Intervention	p-value	Control	Intervention	β (95% CI)	p-value
Break up sitting time TV/DVD (times/h sitting)	2.39 (2.33,2.45)	2.42 (2.29,2.42)	0.43	2.33 (2.25,2.41)	2.47 (2.38,2.55)	0.14 (0.02,0.25)	0.03
Break up sitting time PC/ games console (times/h sitting)	2.19 (2.13,2.25)	2.20 (2.14,2.26)	0.80	2.11 (2.04,2.19)	2.24 (2.17,2.32)	0.13 (0.02,0.24)	0.02*
Break up sitting time at school (times/school h)	1.71 (1.65,1.76)	1.71 (1.66,1.76)	0.92	1.67 (1.58,1.77)	1.77 (1.67,1.87)	0.10 (-0.04,0.23)	0.16
Breaks in sitting time/day#	22.62 (21.71,23.53)	22.79 (21.77,23.80)	0.81	22.50 (21.49,23.40)	22.67 (21.71,23.70)	0.17(-1.18,1.52)	0.81
**Personal determinants**							
Attitude	0.74 (0.67,0.81)	0.94 (0.87,1.00)	<0.001	0.90 (0.81,0.99)	1.15 (1.05,1.24)	0.25 (0.11,0.38)	<0.001**
Preference/liking	0.51 (0.44,0.58)	0.69 (0.62,0.75)	<0.001	0.54 (0.45,0.63)	0.72 (0.66,0.83)	0.20 (0.08,0.32)	0.002***
Self-efficacy	0.67 (0.60,0.74)	0.79 (0.72,0.86)	0.02	0.83 (0.72,0.94)	0.79 (0.68,0.90)	-0.04(-0.20,0.11)	0.59
Automaticy	0.11 (0.04,0.19)	0.17 (0.09,0.24)	0.31	0.10 (0.01,0.18)	0.08 (0.00,0.17)	-0.01(-0.13,0.11)	0.84

Unadjusted means and 95% confidence intervals for the baseline and follow-up values of self-reported and objectively measured behaviors of breaking up sitting time and self-reported determinants of breaking up sitting time, the results of independent t-tests comparing intervention and control groups (at baseline), and regression coefficients (beta’s) with 95% confidence intervals as results from multilevel multiple regression analyses (independent models for each behavior) testing the effects of the UP4FUN intervention.

Ω: adjusted for school (as a random effect), gender and baseline level.

# assessed by accelerometer.

* Significant for Belgium β = 0.29 (95% CI 0.10, 0.49), p = 0.003.

** Significant for Greece β = 0.42 (95% CI 0.15, 0.69), p = 0.01.

*** Significant for Greece β = 0.38 (95% CI 0.17, 0.59), p = 0.002.

**Table 4:** Baseline and follow-up values of self-reported screen time behaviors and objectively measured sedentary time.

**Screen time behaviors and total sedentary time**	Baseline screen time behaviors and total sedentary time			Follow-up screen time behaviors and total sedentary time		Intervention effect Ω	
	Control	Intervention	p-value	Control	Intervention	β (95% CI)	p-value
TV/DVD FQ (h/day)	1.64 (1.59,1.69)	1.62 (1.57,1.67)	0.59	1.57 (1.51,1.63)	1.54 (1.47,1.60)	-0.03 (-0.12,0.05)	0.42
TV/DVD 24h recall (h/day)	1.05 (1.00,1.10)	1.04 (0.99,1.09)	0.80	1.06 (1.00,1.12)	1.00 (0.94,1.06)	-0.06 (-0.15,0.03)	0.19
PC/Games console FQ (h/day)	1.16 (1.11,1.22)	1.17 (1.12,1.22)	0.95	1.19 (1.12,1.26)	1.18 (1.12,1.25)	-0.01 (-0.10,0.09)	0.90
PC/Games console 24h recall (h/day)	0.70 (0.65,0.75)	0.69 (0.64,0.74)	0.78	0.73 (0.67,0.80)	0.75 (0.69,0.82)	0.02 (-0.08,0.12)	0.70
Total sedentary time (h/day) #	8.65 (8.50,8.80)	8.76 (8.60,8.93)	0.34	8.55 (8.31,8.70)	8.44 (8.28,8.59)	0.11 (-0.11,0.33)	0.34

Unadjusted means and 95% confidence intervals for the baseline and follow-up values of self-reported screen time behaviors and objectively measured sedentary time, the results of independent t-tests comparing intervention and control groups (at baseline), and regression coefficients (beta’s) with 95% confidence intervals as results from multilevel multiple regression analyses (independent models for each behavior) testing the effects of the UP4FUN intervention.

Ω: adjusted for school (as a random effect), gender and baseline level.

# assessed by accelerometer.

### Ethics statement

The study adhered to the Helsinki Declaration and the conventions of the Council of Europe on human rights and biomedicine [36]. All participating countries obtained ethical clearance from the relevant ethical committees and ministries. The following ethical committees gave their approval to the study:

Belgium: Medical Ethics Committee of the University Hospital Ghent

Germany: State Medical Chamber of Baden-Württemberg

Greece: Bioethics Committee of Harokopio University

Hungary: Scientific and Ethics Committee of Health Sciences Council

Norway: National Committees for Research Ethics in Norway

Written parental consent was required for the pupil’s participation in the study in all countries except Belgium, where passive informed consent was allowed. The participating parents in all countries except Belgium provided their written consent through their child’s consent form, thereby also agreeing to the participation of one of the parents. In Belgium where passive informed consent was used, the participating parents consented by filling in and returning the parent questionnaire. No verbal consent was asked. The ethics committees/IRBs approved this consent procedure.

## Results

The study sample consisted of 49% boys, and the overall mean age was 11.2 years. In Belgium 706 children participated; 47% boys. In Greece, Hungary, Norway and Germany the numbers were 701 children (45% boys), 635 (50% boys), 554 (50% boys) and 551 (53% boys) respectively.

### Differences at baseline

When comparing intervention and control groups at baseline, no difference was observed for the behaviors, and only few significant differences in determinants (in 8 out of 48 variables) for screen time and breaking up sitting time between the intervention and control group at baseline (Tables 1–3).

### Effects of UP4FUN on TV/DVD watching and use of computer/games consoles (primary outcome) and determinants (secondary outcome)

No significant differences between intervention and control group were observed for self-reported TV/DVD or computer/game console time, neither based on frequency nor on 24h recall data at post-test adjusted for school and baseline (Table 4). The only significant effect of the intervention regarding potential determinants for watching TV/DVD was found for self-efficacy (I find it hard NOT watching TV/DVD), (β = -0.16 (95% CI -0.27,-0.03))—i.e. the intervention group was more likely to agree that it was hard to not watch TV/DVD (Table 1). For two of the determinants for TV/DVD watching (knowledge, attitude) there were significant effects observed in favor of the intervention group in Hungary and Norway (Table 1). For one determinant for computer/games console use (attitude) there was a significant effect observed in favor of the control group in Hungary (Table 2).

### Effects of UP4FUN on breaking up sitting time (primary outcome) and determinants (secondary outcome)

No significant differences were observed for self-reported breaking up sitting time during TV/DVD watching, computer/games console use or at school (Table 3). For breaking up sitting time during computer/games console use, there was a significant effect in favor of the intervention group in Belgium (Table 3). At post-test, the intervention group reported better attitudes towards (β = 0.25 (95% CI 0.11, 0.38)) and preferences/liking for (β = 0.20 (95% CI 0.08, 0.32)) breaking up sitting time compared to the control group (Table 3). For two personal determinants (attitude, preference/liking) there were significant effects in Greece, also in favor of the intervention group (Table 3).

### Effects of UP4FUN on objectively measured sedentary time and breaks in sitting time (primary outcome)

Objectively measured total sedentary time (Table 4) and number of breaks in sitting time per day (Table 3) were not significantly different between the intervention and the control group.

## Discussion

The UP4FUN intervention is among the first school-based and family-focused interventions aimed at reducing and breaking up sitting time related to screen viewing behaviors and with a focus on both school and home environments. We did not find consistent or substantial effects—neither in objective nor in self-reported measures. The data did, however, show a positive effect of the intervention on the attitude towards and preferences/liking for breaking up sitting time (self-reported). This may indicate that the UP4FUN intervention did achieve some changes and may suggest that the children became more aware of the fact that breaking up sitting time is potentially important, and also had gained more positive attitudes towards actually doing so. A study in Sweden by Magnusson et al. [37] found that a belief among children in their own ability to affect their own health was a promising outcome of a community based intervention. However, these changes in beliefs did not result in behavior changes in the present study. Because the items used to assess breaks were newly developed, and these items did not show good test-retest reliability (S1 Appendix), the results regarding breaking up sitting time should be interpreted with caution.

No effects of the intervention were observed on total self-reported screen time, neither on TV/DVD watching nor computer/games console use. UP4FUN included settings both at school and home, involving the whole family, but still did not succeed in reducing sitting time. Possible explanations for this lack of effect may be that, despite efforts to design the intervention according to the intervention mapping protocol [22,23], the intervention may just not have been powerful enough. Secondly, preliminary process evaluation results suggest that the intervention was not implemented completely in all schools, and that there were large differences in implementation across the five countries [38]. It has previously been reported from other studies that school-based interventions are often implemented to a low to moderate degree [39,40]. Thirdly, the family intervention components may not have reached all the parents and family environment, and other channels (e.g. community intervention strategies) are probably needed in addition to involve parents in such interventions. Overall, the intervention did not show effectiveness within the short implementation period of six weeks. Two meta-analyses have stated that in longer lasting interventions, the reduction in screen time was small [17,18]. Marshall et al. also point out that there may be large difficulties in changing sedentary behaviors that have a strong habitual component [41].

One determinant (self-efficacy) for watching TV/DVD was affected by the intervention (I find it hard NOT watching TV/DVD). The intervention children were more likely to agree that it was hard not watching TV/DVD. This could be due to the fact that children in the intervention group tried to limit TV time and by doing this found out that not watching TV is indeed difficult.

In UP4FUN we found a few country differences that may be due to cultural differences. In Belgium, we found a significant intervention effect i.e. children were more likely to break up sitting time during PC/games console usage after the intervention than the control children. This was not found in the total sample or in the other countries, and might be due to the fact that breaking up sitting time (movement breaks) had already reached the teachers of primary schools in Belgium some time before UP4FUN, but the teachers did not really know how to implement them. UP4FUN gave them specific tools on how to do it. So given the already positive attitude among Belgian teachers, this was only a small step. A process evaluation was performed to see how rigorously UP4FUN was implemented in each country. Regarding the significant country-specific determinants of screen time behaviors and breaking up sitting time, these issues are most likely explained by how UP4FUN was implemented, and will be addressed in the process evaluation (paper in preparation).

### Strengths and limitations of the study

Strengths of the present study include the systematic development of the intervention and the large multinational sample of children from different regions across Europe as well as the application of a standardized data collection protocol across the different countries. The cluster randomized trial with pre- and post-test represents a strong design. Furthermore, the study included self-reported behavioral data and self-reported presumed determinant data, as well as objective measurements of total sedentary time and breaking up sitting time (breaks). The self-reported measures were tested for reliability in separate studies [30] (S1 Appendix).

The study also has limitations. Since there were no validated instruments available on assessing self-reported breaking up sitting time and its determinants in children, these items were developed for this study and the determinant items showed poor to moderate test-retest reliability values (S1 Appendix). The quality of the instruments may be a major limitation, since consistency and reproducibility of measurement instruments concern the stability of the results. It may therefore be that the lack of effect was partly due to the possible lack of sensitivity of the used measurement instruments to detect subtle changes. Further, complete and valid accelerometer data was only available in 150 out of the 584 children who were selected for accelerometer measures. Therefore, the lack of significant intervention effects should be interpreted with caution. One of the aims of the project was to reduce and break up sitting time, but accelerometers are no gold standard for measuring sitting and cannot distinguish between sitting still and standing still. Screen time behaviors were based on self-report, and might be biased by social desirable answers and recall bias. Convenience samples of schools in the participating countries were chosen, and therefore we cannot assume representativeness, limiting the generalizability of the results.

## Conclusion

The UP4FUN intervention did not show significant effects in children’s self-reported screen time or objectively assessed sitting time, but it significantly improved attitudes towards and preferences/liking for breaking up sitting time. Overall, these results do not warrant wider dissemination of the present UP4FUN intervention.

## Supporting Information

S1 CONSORT Checklist (DOCX)

S1 Appendix Formulations of the child questionnaire items with response alternatives including test retest reliability. (XLSX)

S1 Protocol Trial Protocol. (DOC)
