# Supplementary figures and images for: Evaluation of the UP4FUN Intervention: A Cluster Randomized Trial to Reduce and Break Up Sitting Time in European 10-12-Year-Old Children
Source: PLoS One. 2015 Mar 31;10(3):e0122612. doi: 10.1371/journal.pone.0122612 (PMC4380348; doi:10.1371/journal.pone.0122612)

**CONSORT checklist**


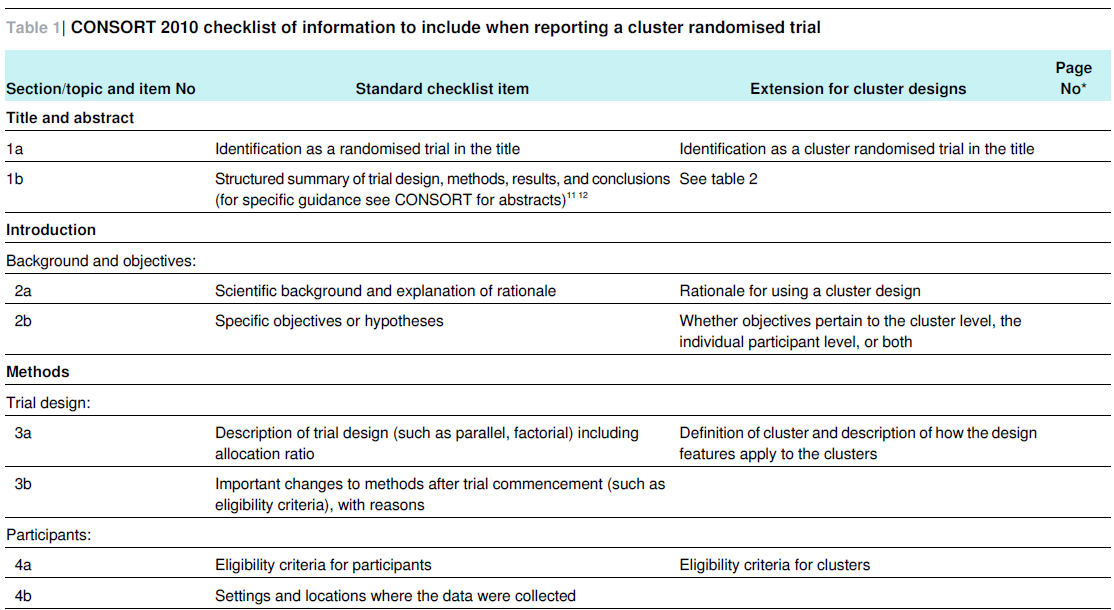

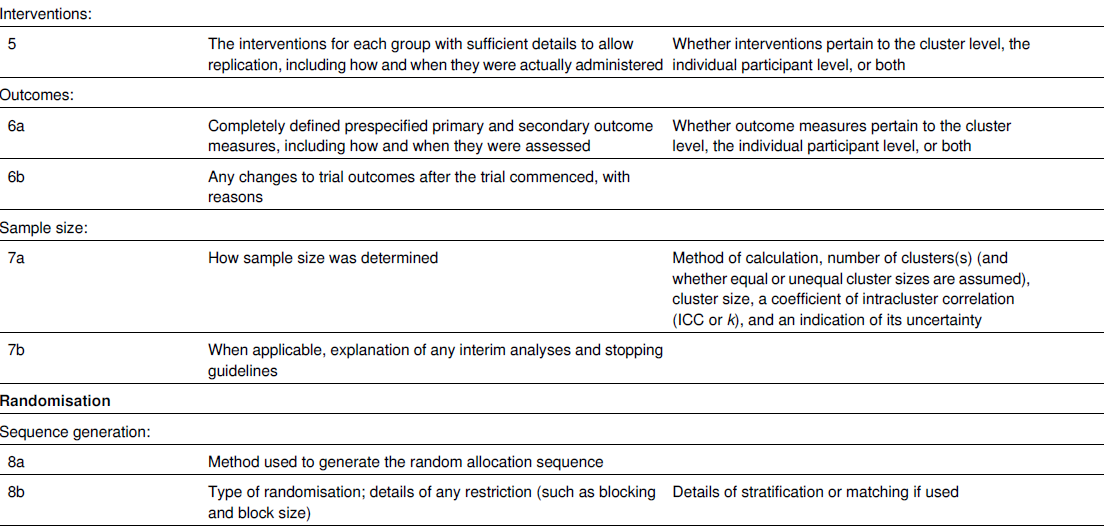

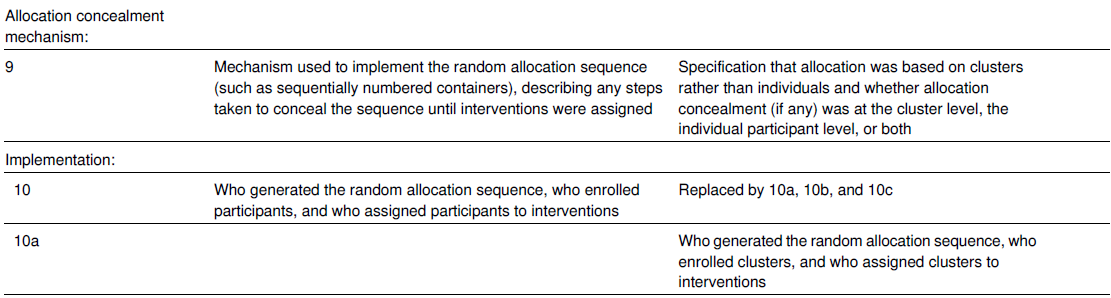


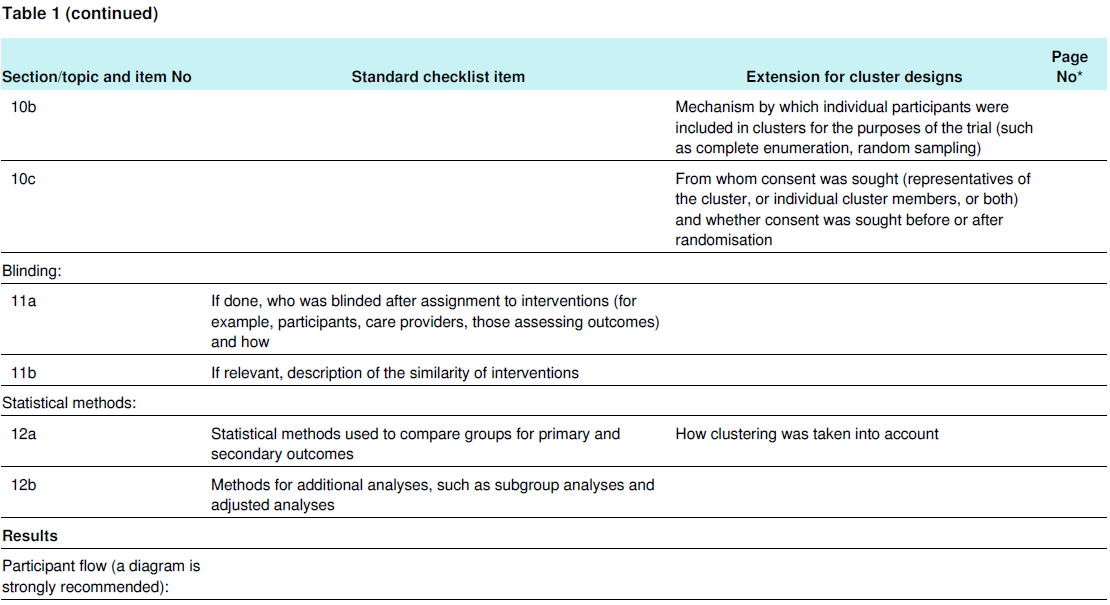

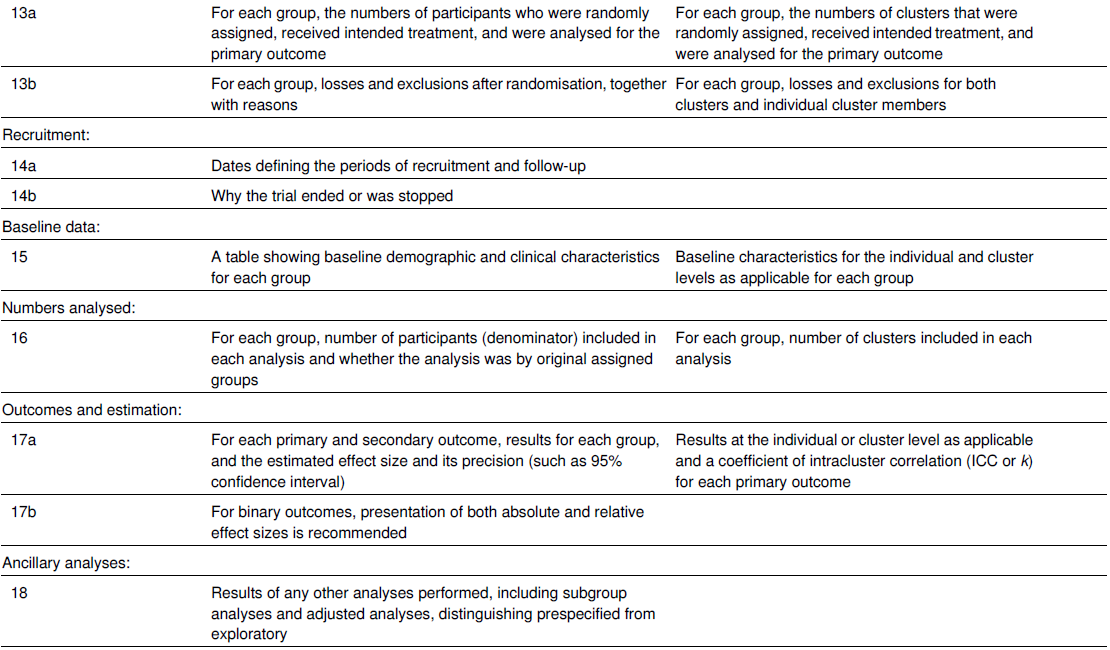

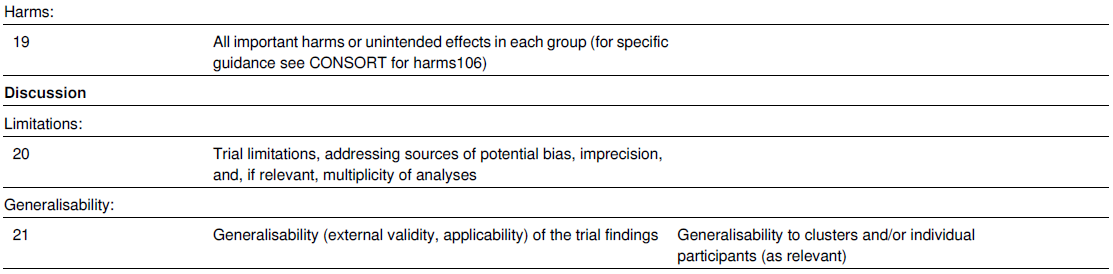


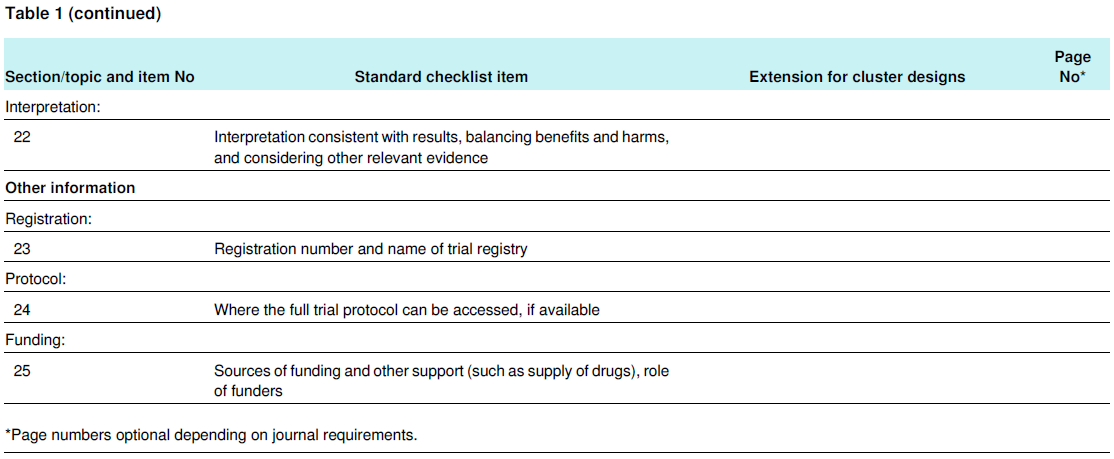

Supplement: S1 CONSORT Checklist — (DOCX) [file pone.0122612.s001.docx]
