# Supplementary material for: Evaluation of the UP4FUN Intervention: A Cluster Randomized Trial to Reduce and Break Up Sitting Time in European 10-12-Year-Old Children
Source: PLoS One. 2015 Mar 31;10(3):e0122612. doi: 10.1371/journal.pone.0122612 (PMC4380348; doi:10.1371/journal.pone.0122612)
Supplement: S1 Protocol — (DOC) [file pone.0122612.s003.doc]

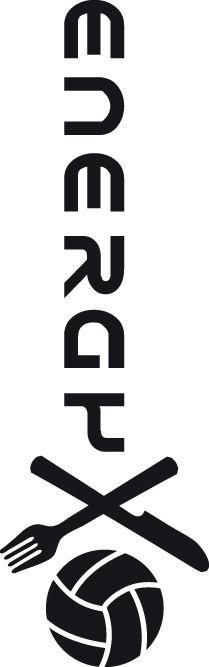
 Trial Protocol

230811

Elling Bere, WP9 leader

**Appendix**

1. Description of Work WP9
   - included below
2. Letter to schools/principals
   - file: *Recruitment letter schools WP9.doc*
3. Letter to parents (including parental consent)
   - file: *Information letter for parents.docx*
4. Child questionnaire
   - file: *WP9 Child Q FINAL 230811.doc*
5. Parent questionnaire
   - file: *WP9 Parent Q FINAL 230811.doc*
6. Child and parent process evaluation
   - File: *WP9 Parent Q II FINAL 171011 NL.doc*
   - File: *WP9 Child Q II FINAL 171011 NL.doc*
7. Teachers process evaluation
   - File: *ENERGY Post process eval Teacher FINAL 141011.doc*
   - File: *ENERGY Post process eval Control school teachers FINAL 141011 (2).doc*
   - File: *ENERGY Process evaluation Teacher logbook FINAL 190911 (3).doc*
   - File: *ENERGY Process evaluation Evaluation teacher training UP4FUN FINAL 010911 (2).doc*
8. Audit instrument
   - file: *ENERGY_AU_FINAL WP9 230811.docx*
9. School management questionnaire
   - file: *ENERGY_SMQ_FINAL WP9 230811.docx*
10. Overview of the intervention
    - file: *ENERGY Table overview intervention 050711.doc*
11. Information and explanations to be given to the children before the fill in the questionnaires
    - file: *WP 9 Explanation UP FRONT in class.doc*
12. Accelerometer protocol paper WP7
    - file: *Yildirim et al 2011.pdf*
    - file: *Yildirim et al 2011 brochure.pdf*
    - file: *Ylidrim et al 2011 diary.pdf* (optional)

**Concept**

Research has shown that the number of children affected by overweight and obesity is now rising at more than 400,000 a year and already affects almost one in four children across Europe.

Overweight and obesity are the result of health behaviours related to nutrition and physical activity causing a positive energy balance; when energy intake through nutrition exceeds energy expenditure, a positive energy balance will result to weight gain. It is less clear which specific dietary, sedentary and physical activity behaviours contribute to excess weight gain in different populations.

ENERGY is a unique project, which for the first time will systematically assess modifiable determinants of energy-balance health behaviours and address the promotion of these behaviours in a European-wide setting.

The main objective of ENERGY is to build a school based, family involved intervention scheme for the promotion of health behaviours that contribute to obesity prevention in children aged 10-12 years old across Europe.

The objective of WP9 is to (1) Conduct a process and outcome evaluation of the intervention, and (2) translate the evaluation results into practical input for WP10 (see Appendix 1).

The intervention scheme was made as part of WP8, and specifically aims at reducing sitting time in school and at home (with special emphasis on TV and PC/electronic games).

The present protocol describes the background and practical procedures for the evaluation study that will be carried out during the fall in 2011 by partners in five different European countries; Belgium, Germany, Greece, Hungary and Norway.

The ENERGY project is supported by the European Commission and is funded by the Seventh Framework Programme (CORDIS FP7) of the European Commission, HEALTH.

**Ethical approval**

All participating countries will collect data among children and their parents. The parents will be fully informed about the contents and the aims of the study and will be asked for consent for the participation of their child in the evaluation study (i.e. to participate in the measurements).

The pupils will complete self administered questionnaires, which aims to identify their sedentary behaviours with determinants, as well as a few physical activity and dietary habits. Additionally, measurements on a subsample of the schoolchildren’s sedentary behaviour and light to vigorous physical activity level will be measured by accelerometers. The parents will also complete a self administered questionnaire related to their own and their child’s nutritional and physical activity behaviours.

This project will in all aspects adhere to the Helsinki Declaration and the conventions of the Council of Europe on human rights and biomedicine. Prior to initiating the project, ethical clearance will have to be obtained from the relevant ethical committees and ministries in all participating countries. Furthermore, research permission, depending on considerations on local and national level, might have to be obtained from local school authorities (local school boards and/or headmasters).

**Sampling procedure**

***Study design***

In order to evaluate the effectiveness of the intervention, the intervention will be tested in a pre-test post-test design including an intervention and a control condition. The control condition will not implement the intervention, but will continue the usual school curriculum. Schools will be the level of randomisation, within each country, in order to prevent contamination of intervention activities.

From each country at least 10 schools with at least 2 classes each (25 children per class on average) will participate in the evaluation study, resulting in total in about 2500 participating children (500 per country). Power calculations based on data from the WP7 cross sectional survey indicate that this sample size is sufficient to detect a 20% decrease in total screen time.

***School sample***

A convenient sample of at least 10 schools in each country will be selected; i.e. schools close to the University or in other convenient areas. In order to be included, each school must have at least 50 10-12 year olds to result in a study sample of at least 500 pupils in each country. The schools will be paired according to size (and also sex and socio economic status if necessary and possible) in order to get similar intervention and control groups. Then one school in each pair will be randomly drawn to the intervention group, and the remaining school will be the control school, by project co-ordinator (VUmc Amsterdam; a partner that will not conduct this evaluation study).

***Study sample***

The study population will be all pupils from the two grade levels that include the majority of pupils born in years 1999 and 2000 (most will be 10-12 year old during fall 2011), and one of their parents. At least two classes per school (one per grade level), with 25 pupils/class should be included in each participating school. All pupils within included classes are invited to participate. However, student participation rate will not be 100%, and over-sampling is needed. Based on the experiences from the cross sectional study in WP7, in which pupil participation rate varied largely with country, each country has to over-sample country specific in order to reach a study sample of 500 (defined by pupils responding to both the pre and the post questionnaire survey).

Over-sampling should firstly be done by adding more classes per school. It is important to always include similar number of classes per school from the two different grade levels included in the study in order to not alter the age level between intervention and control schools. Secondly, add more schools. It is important to always include schools in pairs, in order to have a similar number of intervention and control schools.

In most countries it appears that including four classes per school (in ten schools) is needed in order to reach a final study sample of 500.

**Data collection**

***Recruitment of schools***

The initial contact with the school will be a phone call and a formal letter to the headmaster of the sampled school (Appendix 2). The letter describes the aim of the overall ENERGY project and the purpose of the intervention evaluation study in WP9. If the school does not respond in time, a follow-up phone call will be made.

***Recruitment of pupils and parents***

Following the acceptance of the school to participate in the survey, parents will receive a letter explaining the purpose of the survey and will be asked for consent for child participation in the study. The parent letter is included as Appendix 3. This letter will be brought home by the child and returned by this child back to school. Only pupils with parental consent will participate in the evaluation study. Consent for parental participation is not needed as parents consent by answering the parent questionnaire.

*Ethical approval*

Every country must have obtained the ethical approvals as needed for their country before recruitment of parents and children can start (i.e. before the letter to the parents is sent out).

***Questionnaire surveys***

UiA will conduct the design of the questionnaires and provide the final questionnaires in English (as pdf documents) and pre-coded SPSS-files for entering the data. Each country/partner is responsible for translation into native language and printing (and data entering, see below) of all questionnaires to be used.

*Pupil and parent questionnaires*

Participating pupils and parents will reply to two questionnaire surveys. The pre-test child and parent questionnaire will include a selection of items from the WP7 questionnaires, as well as a few new items where needed. The post test child and parent questionnaires will in addition to the items included in the pre test questionnaires also include process evaluation items (Appendix 6). All new items will first be written in English, then translated to the five languages and back-translated. The questionnaires in English are included as Appendix 4 and 5.

*Teachers’ process evaluation*

A process evaluation will also be conducted during/after the intervention among relevant teachers/school boards (Appendix 7).

*School characteristics*

In order to assess school characteristics of the participating schools relevant items from the school management questionnaire and audit instrument from WP7 will be used (Appendix 8 and 9).

***Accelerometer measurements***

Because of the intervention’s strong focus on sedentary behaviour we will also objectively record sedentary time (and physical activity) with accelerometers in a subsample of about 20% (i.e. 100 pupils per country). Selection of sub sample: Randomly select 3 of the intervention schools and 3 of the control schools, and then randomly select one class/school in the highest class level participating (i.e. 6^th^ grade in most countries, 7^th^ grade in Norway). All children with consent in that class will be asked to participate. If this will not give about 100 pupils, new schools/classes will be selected as above, and always in pairs of two (intervention and control).

**Intervention implementation**

The intervention, primarily aiming at reducing sitting time in school and at home (with special emphasis on TV and PC/electronic games), will last for 6 weeks in the fall semester of 2011 in the intervention schools. The teachers involved will be given training (1.5 hours) as week 0 of the intervention, and completed materials to be used are provided by project workers. During the project period teachers are requested to spend one school hour (45 minutes) to teach a pre-planned lesson each week for 5 weeks. Weekly newsletters (6 in total) for the parents and pre-planned homework for the children will be handed out by the teacher. An overview of the intervention is included as Appendix 10.

**Field procedures**

***Time needed***

Preferably 10 participating schools will provide 500 pupils participating in both pre- and post surveys. A team of two project workers should preferably visit one school/day to perform the data collection. Mondays can not be used due to the recall questions in the questionnaire, and a period of 3 weeks should be planned to conduct the data collection at both pre- and post surveys. Involving more project staff could reduce the data collection time periods. However, due to the accelerometer measurements, and the limited numbers of accelerometers available (ca 60 per country), two weeks are seen as an absolute minimum.

The evaluation study will take place within the fall semester of 2011. The implementation of the intervention will last for 6 weeks. The pre test has to be finished before the intervention implementation starts, and the post test has to start as soon as possible after the end of the intervention implementation. A total of three weeks is allocated to both the pre- and the post tests, giving a full duration of the intervention study of 12 weeks. Regarding slightly different semester schedules in the different countries, each country has to plan their own timing. Most countries have a one week break within the semester, giving a total time frame for the evaluation study of at least 13 weeks.

***Procedures at the survey day***

At least two project workers have to be at school at the survey day in order to conduct the child questionnaire survey, initiate the accelerometer measurements, hand out the parent survey, school management survey and teachers process evaluation survey, and to fill out the audit instrument. The survey day will take place Tuesdays-Fridays. Extra questionnaires should be brought to the school if pupils appear with parental consent on the survey day.

*Child survey (BOTH PRE- AND POST TEST)*

The time needed for the child survey is one school hour (45 minutes max). At least one researcher will be present in the class while the pupils respond to the questionnaire. The pupils not taking part in the study will do other work (make an appointment with teachers ahead of the survey day). Explain the purpose of the study to the children. Make sure that children do not mix up the questionnaires because they are coded individually. Distribute the child questionnaire. It is of outmost importance that every child gets a questionnaire with their unique ID number (but no names will be written on the questionnaire). The pupils will be told that it is important to give honest answers, that the answers will not be judged, that all their answers will be treated secretly, that their parents, teachers and class mates will not be informed about their answers and that they can withdraw any moment during the study. Read out the standardized instruction form to the children on how to fill in the questionnaire in sections (front page of questionnaire), and explain the most important variables (see APPENDIX 11). When the children are finished, collect the questionnaires. The children will continue with other work until the school hour is finished. During the school hour, every child gets an envelope with the parents’ questionnaire to give to their mother or father. This questionnaire should be returned to the teacher before a specific date (within one week). It is of outmost importance that also the parent’s questionnaire and envelope contains the child’s unique ID number (and no names).

Some pupils may have problems in understanding some questions. Therefore carefully explain what is meant by the main variables asked for in the questionnaire (APPENDIX 11). Researchers can also help pupils individually if major problems occur, but are urged to keep interaction to a minimum to reduce between country variations.

If children are absent, leave the questionnaire together with an empty return envelope with the teacher. The child can fill in the questionnaire within the following week, and give it to the teacher (in the closed envelope), for return to the researcher together with the parent questionnaires.

*Child accelerometer measurement (BOTH PRE- AND POST TEST)*

The accelerometer measurements will follow the protocol for the accelerometer study in WP7 (Appendix 12). Those children that are selected to take part in the accelerometer study will during the same school day be given the accelerometer, and be instructed how to use it. The accelerometers will be worn for 6 consecutive days (in order to cover at least 2 school days and 2 weekend days). Project staff will collect the accelerometers at school the following week. Accelerometers have to be initialized and prepared (checking battery) according to instructions of the manufacturer before handing out in schools. Project staff has to attach the accelerometer using an elastic belt at the participants' hip, near spina iliaca anterior superior. The accelerometer has to be initialized to start the day after attached to the body of the participant. Exactly the same accelerometer settings as used in the accelerometer study in WP7 will be applied (Appendix 12). Information about accelerometer use will be given to schoolchildren and at the end of the information session, accelerometers will be handed out. Additionally, children and parents will receive a brochure about accelerometer use. Usage of a diary for recording when the accelerometer is taken off and why is optional and it is up to every partner to decide what is best for greatest compliance (the diary is included in APPENDIX 11). Teachers will also be informed about the procedures and asked to remind the children to wear the devices every day. Children will bring the device back to school and hand it to their teacher. Accelerometers will be collected from schools the day after. They will be prepared for the next school, i.e. charging, downloading data, initializing. Raw data (dat. - and csv-files as separate files for each child) has to be coded and then delivered to UiA using Dropbox. Accelerometer data files will be cleaned and analysed in Norway (UiA).

*Parent survey (BOTH PRE- AND POST TEST)*

While conducting the child survey every child gets one envelope to be given to their parents, including the parental questionnaire and an empty envelope for return to school. It is of utmost importance that this questionnaire includes the corresponding child ID number. No names will be written anywhere. Parents will send the questionnaires in the closed envelope back to school. It is important that it is the same parent that responds to both the pre- and the post test.

*School characteristics (PRE TEST)*

The observations (audit instrument) of the school environment will be conducted by a researcher on the survey day (pre test only). On the survey day the school management questionnaires will be given separately to each headmaster. On the day of receiving the parent questionnaires the researcher will also receive the completed school management questionnaire.

*Teachers process evaluation (POST TEST)*

On the survey day the teachers involved in the intervention will be given process evaluation questionnaires. On the day of receiving the parent questionnaires the researcher will also receive the completed process evaluation questionnaires from the teachers, together with the logbooks.

*Return of parent, school management and teachers questionnaires and accelerometer devices*

Six days after the survey day the researcher should visit the school again to collect the parent, school management and teachers questionnaires and the accelerometer devices. A second round of collection might be needed, and/or questionnaires arrived later might be sent by mail.

***Training of research staff***

Main field project staff in each country will be trained in the ENERGY meeting in Athens April 13-15 2011. Remaining field project staff will be trained by main country responsible within each country.

**Data management**

***Identification***

In order to merge data from the different surveys, it is of utmost importance that the different data sources are marked with a unique ID for every participant. Parents have the same ID number as their children. Every country/partner has to keep an ID-key, i.e. a list of participating names including every child’s unique ID number. This list has to be kept separately from the data files.

The unique ID numbers (always 7 digits) follow this code:

- Country number: 1 digit (Belgium = 1, Germany = 9, Greece = 2, Hungary = 3, Norway = 5).
- School number (within country): 2 digits (intervention schools will be given numbers from 01-10, control schools will be given numbers from 11-20)
- Class level: 2 digits (5, 6 or 7 – corresponding to grade level, and 1, 2 etc for separating classes in same grade level (i.e. 51 is grade 5 class A, 52 is grade 5 class B)
- Child number: 2 digits

Example ID 9026213: 9=Germany, 02=German school no. 2, intervention school, 62=6^th^ grade, class B, 13=pupil no. 13 in that class.

The ID will be the same for the child and the parent questionnaires and child accelerometer data. An audit instrument and school management questionnaire will be given an ID based on country and school. The teacher process evaluation questionnaires will be given an ID based on country, school and class (i.e. one teacher per intervention class).

***Data entry***

Each partner/country is responsible for entering all collected data into SPSS. UiA will provide pre-coded SPSS templates, including definition of values to be given each answering alternative in all questions for all different surveys, in which the data must be entered. Each country/partner decides whether to manually insert the data, or to do it by optical scanning.

For all questions where only one tick is allowed (i.e. most questions) the coding is always like this: 1st answering alternative=1, 2nd answering alternative=2, 3rd answering alternative=3, 4th answering alternative=4 and so it goes. Missing is always coded -9. When two neighbour boxes are both ticked, or a tick is in between two boxes, choose the most conservative estimate (i.e. the alternative closest to the middle). If the two alternatives are both equally close to the middle choose one of them at random. If three or more ticks, or two ticks that are further apart than the closest box, answer is coded as missing.

For questions where multiple ticks are allowed, every answering alternative is a separate variable where a tick is coded 1 and not a tick is coded 0. However, if there are no ticks in any of the answering alternatives all variables/answering alternatives will be coded -9 (missing).

For questions with written numbers just fill in the number written. NB. there are two variables for each sleep question: (a) Hours and (b) Minutes.

Raw data (as non-personal identifiable SPSS files) will be sent UiA by e-mail as soon as they are ready.

***Data cleaning***

Each partner/country is responsible for cleaning their data.

To ensure that the data has been entered correctly in the child and parent questionnaires a total of 5% of all hand inserted child and parents questionnaire will be entered a second time (in a separate file) (not needed if the data are scanned). If >3% of the data differs, all questionnaires have to be re-entered. Also 20% of the audit instrument and the school management questionnaires will be re-entered, and differences will be assessed.

All variables must be checked in order to certify that all values are within the defined ranges for the separate variables. If values are outside min-max range the questionnaire must be checked and values be replaced with the correct values.

Cleaned non-personal identifiable SPSS files will be sent UiA by e-mail.

***Data recoding and restruction***

This will be done by UiA based on the recoding and restruction conducted in WP7. A final SPSS file will be sent to all partners, and also VUmc for central data storage.

**Data interpretation**

The outcome evaluation will be based on a set of multilevel (i.e. adjusted for school) regression analyses on differences between intervention and control groups on post-test values, as recommended by Twisk and Proper (*J Clin Epidemiol* 2004, **57:**223-228), adjusted for baseline values and sex. Separate similar analyses will be conducted for parents (only including parents answering both the pre-test and post-test questionnaire). For the process evaluation descriptive analyses will be presented.

**APPENDIX 1 – From Grant agreement Annex I - “Description of Work”**
